# ForDigitStress: presentation and evaluation of a new laboratory stressor using a digital job interview-scenario

**DOI:** 10.3389/fpsyg.2023.1182959

**Published:** 2023-06-19

**Authors:** Linda Becker, Alexander Heimerl, Elisabeth André

**Affiliations:** ^1^Chair of Health Psychology, Department of Psychology, Friedrich-Alexander-Universität Erlangen-Nürnberg, Erlangen, Germany; ^2^Chair for Human-Centered Artificial Intelligence, Institute of Computer Science, Universität Augsburg, Augsburg, Germany

**Keywords:** digital stress, job interview, stress induction, stress test, salivary alpha-amylase, cortisol, threat, challenge

## Abstract

**Introduction:**

Since the COVID-19 pandemic, working environments and private lives have changed dramatically. Digital technologies and media have become more and more important and have found their way into nearly all private and work environments. Communication situations have been largely relocated to virtual spaces. One of these scenarios is digital job interviews. Job interviews are usually—also in the non-digital world—perceived as stressful and associated with biological stress responses. We here present and evaluate a newly developed laboratory stressor that is based on a digital job interview-scenario.

**Methods:**

*N* = 45 healthy people participated in the study (64.4% female; mean age: 23.2 ± 3.6 years; mean body mass index = 22.8 ± 4.0 kg/m^2^). Salivary alpha-amylase (sAA) and cortisol were assessed as measures for biological stress responses. Furthermore, perceived stress was rated at the time points of the saliva samplings. The job interviews lasted between 20 and 25 min. All materials, including instructions for the experimenter (i.e., the job interviewer) and the data set used for statistical analysis, as well as a multimodal data set, which includes further measures, are publicly available.

**Results:**

Typical subjective and biological stress-response patterns were found, with peak sAA and perceived stress levels observed immediately after the job interviews and peak cortisol concentrations 5 min afterwards. Female participants experienced the scenario as more stressful than male participants. Cortisol peaks were higher for participants who experienced the situation as a threat in comparison to participants who experienced it as a challenge. Associations between the strength of the stress response with further person characteristics and psychological variables such as BMI, age, coping styles, and personality were not found.

**Discussion:**

Overall, our method is well-suited to induce biological and perceived stress, mostly independent of person characteristics and psychological variables. The setting is naturalistic and easily implementable in standardized laboratory settings.

## 1. Introduction

Stress is an ubiquitous phenomenon, which is accompanied by the subjective feeling of being stressed as well as a variety of biological stress responses. The biological stress response is complex and involves several stress systems. In acute stress situations, the two most important ones are the activation of the sympathetic nervous system (SNS), which is a part of the autonomous nervous system and the activation of the hypothalamic–pituitary–adrenal (HPA) axis (Fulford and Harbuz, 2005). SNS responses occur instantaneously, and peak levels can be found during and immediately after stressors. The SNS response is associated with the so-called fight-or-flight response (Selye, 1950). It can be assessed by means of several markers, of which one is the assessment of the enzyme salivary alpha-amylase (Nater and Rohleder, 2009). In contrast, HPA axis responses show a slower time course. Usually, peak cortisol concentrations can be found approximately 20 min after the end of typical (e.g., socially evaluative) stressors. Cortisol levels can be easily measured in saliva or blood samples (Vining et al., 1983; Putignano et al., 2001).

The overall stress response is complex and strongly depends on the nature of the stressor (so-called specificity hypothesis; Kemeny, 2003). A further crucial factor is person himself/herself and his/her appraisal of the situation (Lazarus and Folkman, 1984, 1987). Stressors perceived as a threat usually lead to strong biological stress responses in contrast to stressors perceived as a challenge (Kemeny, 2003).

For conducting research on biological stress responses, standardized laboratory stress tests are needed. Popular ones are the Trier Social Stress Test (TSST; Kirschbaum et al., 1993), the socially evaluative cold-pressor test (SECPT; Schwabe et al., 2008; Becker et al., 2019), or the Montreal Imaging Stress Task (MIST; Dedovic et al., 2005). However, some of these stressors have the drawback that they are only partially realistic. Moreover, they are personnel-intensive, and digital implementation is difficult or not feasible in some cases (e.g., when the task is to put your hand in ice water as in the case of the SECPT). Therefore, new laboratory stressors that are more similar to common field stressors, that simulate naturalistic settings, and that can be implemented in an online setting, are needed. Nevertheless, if a setting is transferred to the virtual space, the question remains open whether stress responses are triggered and therefore evaluation studies are needed.

Due to increased digitalization, modern stressors are developing. Since the pandemic, which started in 2019, private and working life often takes place virtually. Online meetings and communication tools are widespread. Not only routine work was shifted to the virtual world, but also less frequent situations such as short-term agreements and negotiations. Job interviews, e.g., are usually—also in the analog world—perceived as stressful and can lead to biological stress responses (Budnick et al., 2019). Digitalization of this setting (i.e., job interviews) has the advantage of being cost-efficient, flexible, and standardizable across many applicants. In a pilot study, we have already proved the feasibility of setting-up a realistic and stressful job interview-scenario in the laboratory (Heimerl et al., 2022a).

In this study, we aimed to develop a standardized laboratory stressor, which was based on a digital job interview-scenario. We here present the protocol of our setting and an evaluation of our procedure. All materials are freely available for other researchers (see 11). Our objective was 3-fold: First, we developed the setting and provided a detailed description of the materials. Second, we conducted an evaluation study to investigate whether our scenario, indeed, induces stress (of the SNS, the HPA axis, and perceived stress). Third, we tested for potential associations between the strength of the stress responses and person characteristics [e.g., age, sex, body mass index (BMI), and psychological variables such as personality traits and coping styles].

## 2. Methods

### 2.1. Participants

Overall, *N* = 52 healthy people, who were non-smokers, participated in the study. Data from *n* = 7 participants had to be excluded from statistical analysis [*n* = 1 had withdrawn his consent and the data has been deleted immediately, *n* = 1 due to technical issues, *n* = 1 inconsistencies in the answer regarding previous job interviews, *n* = 2 non-binary sex, *n* = 2 statistical outliers regarding the variable age (57 and 61 years)]. The final sample size was *N* = 45 (64.4% female; mean age: 23.2 ± 3.6 years, range 18–33 years; mean BMI = 22.8 ± 4.0 kg/m^2^, range: 17.9–37.7 kg/m^2^). The study has been approved by the local Ethics Committee of the Medical Faculty of the Friedrich-Alexander-Universität Erlangen-Nürnberg (protocol number: 21-408-S).

### 2.2. Procedure

#### 2.2.1. General procedure

**Figure 1:**
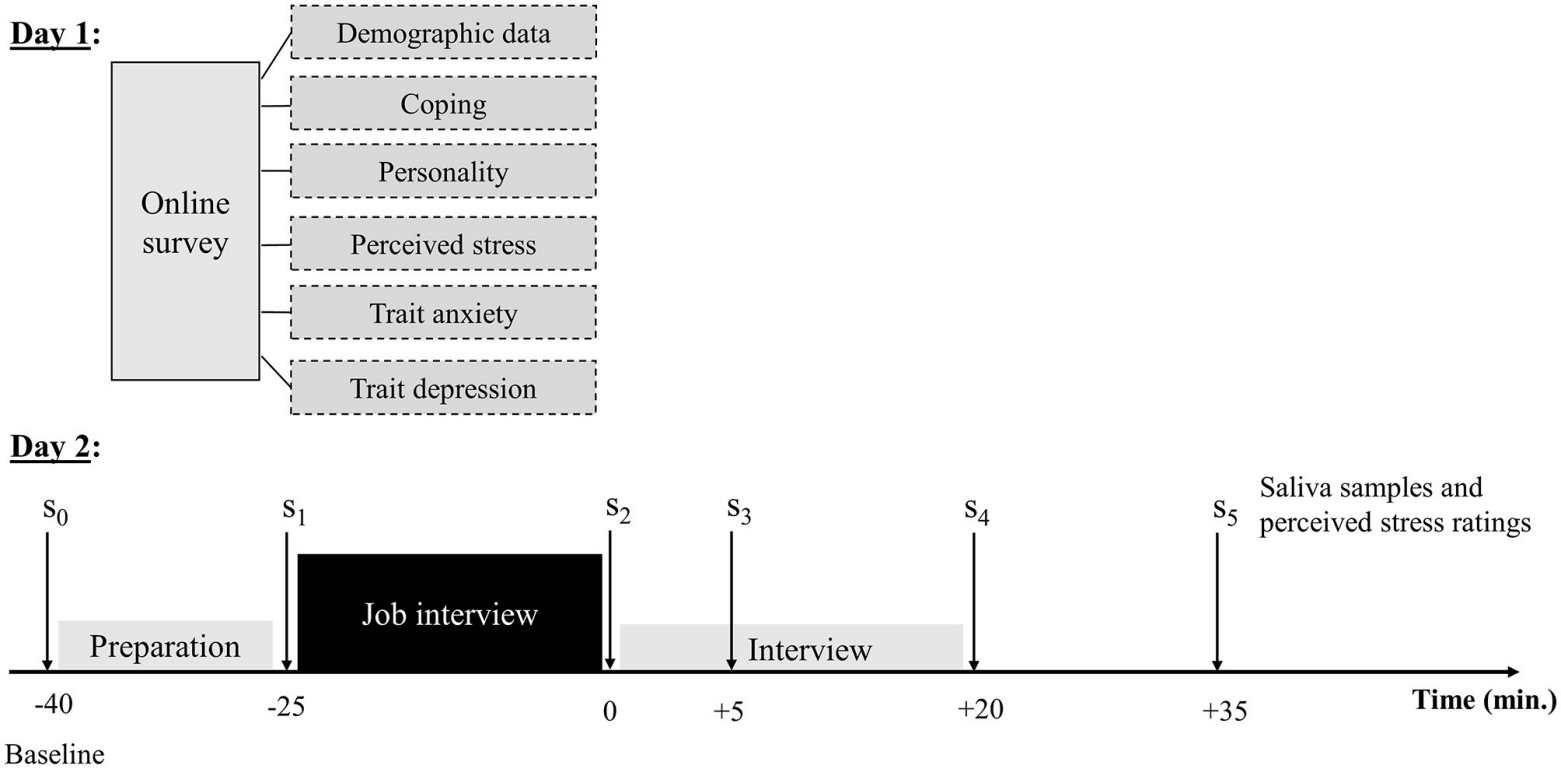
Procedure. The study involved two parts on different days. The online survey on day 1 lasted about 15–20 min, and the laboratory session on day 2 took about 90 min.

The study consisted of two parts (Figure 1). Prior to the actual experiment (day 1), participants sent their curriculum vitae (CV) to the experimenters and filled out an online survey, which included questions about demographic data, their experience with previous job interviews, and psychological questionnaires (e.g., coping styles and personality traits; 2.3). On the day of the experiment (day 2), participants came to our laboratory. First, the saliva collection procedure was introduced, and the first saliva sample (s_0_) was taken. After this, participants were equipped with an eye tracker and a finger-clip pulse sensor (Further recordings; 2.3.4). Then, the participants were asked about their dream job, which they would apply for at the job interview, and they were seated in front of a computer.

The preparation phase lasted about 15 min, and participants were instructed to prepare for the job interview and to take notes if necessary. After the end of the preparation phase, the next saliva sample (s_1_) was taken. The actual job interview started afterward on the computer screen in front of the participants and lasted about 20–25 min (see The job interview-scenario for further details on the job interviews; 2.2.2). The interviewer sat in another room and was hidden from the participant until the job interview started. The job interview was conducted via *Zoom*. Immediately after the job interview, the next saliva sample (s_2_) was taken. After this, participants were interviewed about their experiences and emotions during the job interview (Interviews preceding the job interviews). Further saliva samples were taken 5 (s_3_), 20 (s_4_), and 35 (s_5_) min after s_2_. The time course of the general procedure is shown in Figure 1.

#### 2.2.2. The job interview-scenario

Our aim was to develop a scenario, which is (1) realistic and (2) associated with acute stress responses (perceived and/or biological) for most participants. As described in Introduction (1), we decided to use a digital job interview-scenario based on a setting, which has proven to be perceived as stressful and associated with negative emotions in a pilot study (Heimerl et al., 2022a).

The job interviews were individually adopted for each participant and based on the individual job, for which they decided to apply for. The CV was used as a basis for the interviews as well, and individual questions were asked with regards to the job for which participant applied.

The general structure of the job interviews was as follows:

1. Welcome and introduction
2. Introduction of the applicant (e.g., “*Please introduce yourself”*)
3. Interruption of the introduction (e.g., “*Don't tell me anything that I could read on your CV”*)
4. Questions regarding the employer/company (e.g., “*Why do you want to join this company?”*)
5. Job change explanation (e.g., “*Why are you looking for a new job?”*)
6. Expectations of the applicant (e.g., “*What do you expect from the job?”*)
7. Advertising of the applicant (e.g., “*What qualifies you for this job?”*)
8. Hypothetical situation (e.g., “*How would you react if...?”*)
9. Applicant's future vision (e.g., “*Where do you see yourself in 5 years?”*)
10. Practical task (e.g., “*Sell me this pen”*)
11. Basic knowledge questions (e.g., “*On what date did the Berlin Wall fall?*”)
12. Subject-specific questions (e.g., “*Please translate the following sentence…”*)
13. Questions regarding the applicant's outfit (e.g., “*Why are you wearing this outfit today?”*)
14. Ideas regarding salary and working hours (e.g., “*Are you willing to work overtime?”*)
15. End.

More examples of specific questions, which were asked during the different parts of the interviews, are provided as Supplementary material 1. The order of questions differed among the participants and not every part and not every question was used for all participants. If questions were answered easily, the next part was started. When participants struggled with a question, the interviewer kept on asking.

#### 2.2.3. Interviews preceding the job interviews

Immediately after the job interview (i.e., after s_2_), participants rated whether they perceived the job interview rather as a threat or as a challenge and whether they experienced stress during the job interview. The rating as threat vs. challenge was used for the following analyses.

If participants reported that they felt stressed, they were asked to describe the situation/s in which they experienced stress as accurately as possible. After this, they were interviewed about their emotions during the job interview. They were asked whether they experienced a specific emotion, and if so, they were instructed to report the specific situation in more detail. The following emotions were used: anxiety, shame, sorrow, pride, joy, self-confidence, being alarmed, depression, nervousness, creativity, and luck. Afterwards, participants were asked whether any further emotions were present during the job interviews.

The data from these interviews were used to annotate the data set and create a multimodal stress data set, which is reported in Heimerl et al. (2023); but which was not part of the analyses, which are reported here.

### 2.3. Materials

#### 2.3.1. Psychological variables

Psychological variables were assessed by means of the following standardized questionnaires, which were included in the online survey (day 1):

##### 2.3.1.1. Coping

Coping has been assessed using the German 24-item version of the Coping Inventory for Stressful Situations (CISS; Endler and Parker, 2008; Kälin and Semmer, 2020). The scale measures task-oriented, emotion-focused, and avoidance-oriented coping.

##### 2.3.1.2. State anxiety and depression

Trait anxiety and depression have been assessed using the trait items from the State-Trait Anxiety-Depression Inventory (STADI-T; Laux et al., 2013).

##### 2.3.1.3. Personality

For personality assessment, the short version of the Big Five Inventory (BFI-S; Rammstedt and John, 2005) has been used. The BFI-S scale enables the assessment of the big-five dimensions extraversion, neuroticism, agreeableness, conscientiousness, and openness to experience.

##### 2.3.1.4. Perceived stress

Perceived stress during the last month has been measured using a German translation of the 10-item version of the Perceived Stress Scale (PSS; Cohen et al., 1994; Klein et al., 2016).

#### 2.3.2. Perceived stress during the job interview

During each saliva sampling, participants rated their current level of perceived stress, tiredness, and exhaustion on 10-point Likert scales (Becker and Rohleder, 2019, 2020; Becker et al., 2022b). The anchors were “*not stressed at all*” and “*totally stressed.”* Note that only perceived stress is evaluated in the following due to high intercorrelations.

#### 2.3.3. Laboratory analyses of saliva samples

As described above, salivary alpha-amylase and cortisol were assessed from saliva samples that were collected by means of Salivettes at six-time points (Figure 1). Salivettes were stored at −30°C. Before analysis, they were thawed at room temperature and centrifuged at 2,000 g at 20°C for 10 min. Salivary alpha-amylase was measured with an in-house enzyme kinetic assay using reagents from DiaSys Diagnostic Systems GmbH (Holzheim, Germany), as described elsewhere (Bosch et al., 2003; Nater et al., 2007). In brief, saliva was diluted at 1:625 with ultrapure water, and diluted saliva was incubated with substrate reagent (α-amylase CC FS; DiaSys Diagnostic Systems) at 37°C for 3 min before the first absorbance reading was taken at 405 nm with a Tecan Infinite 200 PRO reader (Tecan, Crailsheim, Germany). A second reading was taken after 5 min incubation at 37°C. An increase in absorbance was transformed to sAA concentration (U/ml) using a standard curve prepared using “Calibrator f.a.s.” solution (Roche Diagnostics). Cortisol concentrations in nmol/l were analyzed using commercially available high-sensitive enzyme-linked immunosorbent assay (ELISA; IBL international). The procedure is described elsewhere (e.g., Becker et al., in press). All analyses were conducted in duplicate. Intra coefficients of variation (intra-CV) were below 10% for both sAA and cortisol.

#### 2.3.4. Further recordings

During the whole session, participants wore an eye tracker (*Pupil Labs*), were videotaped, and movements were recorded using a *Microsoft Kinect 2*. Furthermore, they were equipped with a finger-clip sensor (*iom*) on the non-dominant hand. This data is reported in Heimerl et al. (2023) as part of the annotated multimodal data set.

### 2.4. Statistical data analyses

For statistical analysis, IBM SPSS Statistics (version 29 for Windows) was used. For descriptive statistics, means (*M*) and standard deviations (*SD*) were calculated. Prior to further analysis, sAA and cortisol concentrations, as well as age and BMI, were transformed using the natural logarithm (ln) to achieve a normal distribution. Participants with values that were more than three standard deviations away from the participant's mean were treated as outliers and excluded from further analysis.

To test whether the interviews induced stress responses of the SNS and the HPA axis as well as perceived stress, analyses of variance for repeated measurements (rmANOVAs) were calculated for sAA, cortisol, and perceived stress ratings with the within-subjects factor ‘time' which reflected the 6 measurement time points (s_0_-s_5_). If necessary, sphericity violations (determined by Mauchly's test of sphericity; Mauchly, 1940) were corrected by adjusting the degrees of freedom with the procedure by Greenhouse and Geisser (1959). *T*-tests for dependent samples were used for *post hoc* pairwise comparisons, if needed. Cohen's *d* was used as a measure for effect size.

For the investigation of potential sex differences and differences between participants who experienced the situation as a threat vs. as a challenge, further rmANOVAs were calculated for all three variables (sAA, cortisol, and perceived stress) with sex or threat vs. challenge as a between-subjects factor. Similar analyses were conducted for female participants who reported the use of contraceptives and for participants with the same vs. a different sex than the interviewer.

To test whether the stress responses were associated with ln(age), ln(BMI), number of previous job interviews, time of day, coping, perceived stress during the last month, personality, trait anxiety or depression, Pearson's correlations between these variables and the difference between the maximum sAA, cortisol, and perceived stress level and the value at s_1_ were calculated and tested for significance.

An adjusted alpha level of 0.05/3 = 0.017 was used for all analyses because three main outcome variables (sAA, cortisol, and perceived stress) were evaluated.

### 2.5. Availability of materials

All materials used in the study, a comprehensive overview of the job interview-scenario and the data set which has been used for the analyses within this article, are freely available as Supplementary material 1 or under https://osf.io/5bdyf/. Furthermore, an annotated multimodal data set, which has been created during the study and which contains further modalities (e.g., heart rate, movements, and pupillometric data), is available upon request from http://hcai.eu/fordigitstress.

## 3. Results

### 3.1. Descriptive statistics

The characteristics of the final sample, including the psychological variables, are reported in Table 1 for nominal-scaled variables and in Table 2 for metric variables.

**Table 1:** Sample characteristics for nominal-scaled variables (*N* = 45).

**Variable**		***N***	***%***
Sex	Female	29	64.4
	Male	16	35.6
Same sex as the interviewer	No	24	53.3
	Yes	21	46.7
Smoker	No	45	100
	Yes	0	0
Use of contraceptives (women only, *n* = 29)	No	24	82.8
	Yes	5	17.2
Ethnicity	White	42	93.3
	Asian	1	2.2
	Other	2	4.4
Education	Secondary school level (“*Mittlere Reife*”)	2	4.4
	Vocational diploma (“*Fachabitur*”)	3	6.7
	General qualification for university entrance (“*Abitur*”)	32	71.1
	Bachelor's degree	7	15.6
	Diploma or master's degree	1	2.2
Profession	Student	37	82.2
	Full-time employee	8	17.8
Experience with job interviews	No	8	17.8
	Yes	37	82.2
Job interview rating	Challenge	32	71.1
	Threat	10	22.2
	Neither nor	3	6.7

**Table 2:** Sample characteristics for metric variables (*N* = 45).

**Variable**	**Mean**	**Standard deviation**	**Minimum**	**Maximum**
Age (years)	23.2	3.6	18	33
BMI (kg/m^2^)	22.8	4.0	17.9	37.7
Time of day (hours)	13.4	2.6	9.5	18.2
Number of job interviews (if experience, *n* = 37)	5.0	3.9	1	20
Task-oriented coping (CISS)	30.4	4.4	18	40
Emotion-focused coping (CISS)	22.4	5.0	12	33
Avoidance-oriented coping (CISS)	23.5	6.3	10	34
Perceived stress (PSS)	16.9	6.1	4	28
Extraversion (BFI-S)	3.6	0.9	2.25	5
Agreeableness (BFI-S)	3.4	0.7	1.75	4.75
Conscientiousness (BFI-S)	3.8	0.5	2.25	5
Neuroticism (BFI-S)	3.1	0.8	1.5	4.5
Openness to experience (BFI-S)	3.9	0.6	2.6	5
Trait anxiety (STADI-T)	20.6	5.6	11	34
Trait depression (STADI-T)	18.1	4.7	10	30

BMI, body mass index; CISS, Coping Inventory for Stressful Situations (Endler and Parker, 2008; Kälin and Semmer, 2020); PSS, Perceived Stress Scale (Cohen et al., 1994; Klein et al., 2016); BFI-S, short form of the Big Five Inventory (Rammstedt and John, 2005); STADI-T, trait items from the State-Trait Anxiety-Depression Inventory (Laux et al., 2013).

### 3.2. Biological and perceived stress responses

#### 3.2.1. Salivary alpha-amylase

**Figure 2:**
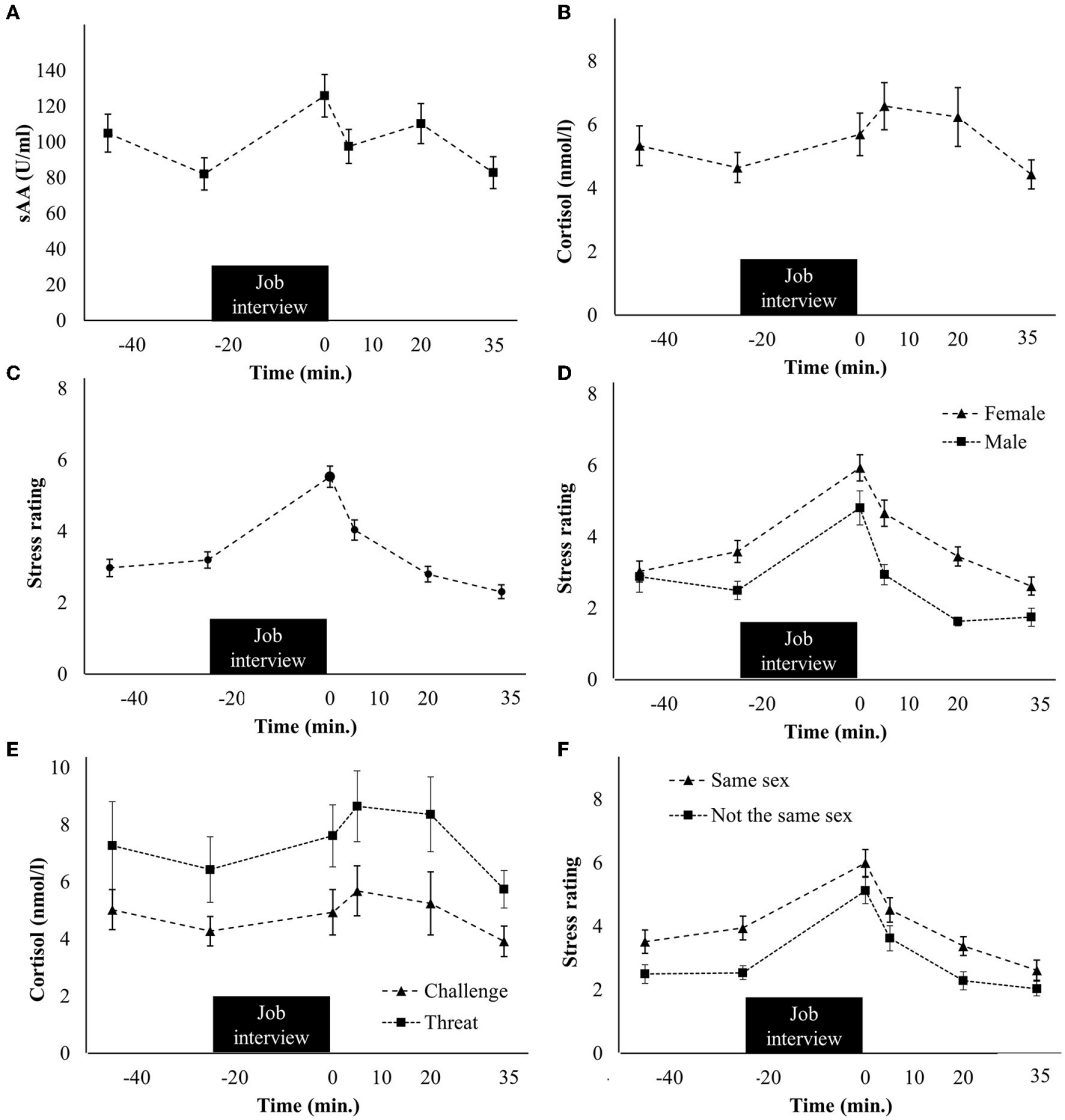
**(A)** Time course of the salivary alpha-amylase (sAA), **(B)** cortisol, and **(C)** perceived stress **(C)** responses, **(D)** sex differences in perceived stress levels, **(E)** cortisol time course for participants who rated the situation as a threat vs. as a challenge, and **(F)** perceived stress levels in dependence on whether the interviewer had the same sex as the participants vs. an opponent sex. Standard errors are shown as error bars.

For sAA, a significant main effect of the factor time was found [*F*_(3.60, 151.27)_ = 6.32, *p* < 0.001, $\eta_{\text{p}}^{2}$ = 0.13], indicating that sAA levels significantly changed during the session. *Post hoc t*-tests indicated that sAA levels were significantly higher at s_2_ (i.e., immediately after the job interview) than before the job interview at s_1_ [*t*_(42)_ = 3.68, *p* < 0.001, Cohen's *d* = 0.80; Figure 2A].

#### 3.2.2. Cortisol

For cortisol, a significant main effect of the factor time was found [*F*_(1.60, 68.89)_ = 4.92, *p* = 0.015, $\eta_{\text{p}}^{2}$ = 0.10], indicating that cortisol levels significantly changed during the session. *Post hoc t*-tests showed that cortisol levels were significantly higher at s_3_ (i.e., 5 min after the job interview) than before it at s_1_ [*t*_(43)_ = 3.41, *p* < 0.001, Cohen's *d* = 0.60; Figure 2B].

#### 3.2.3. Perceived stress

Perceived stress also significantly changed during the session [*F*_(3.54, 155.79)_ = 44.54, *p* < 0.001, $\eta_{\text{p}}^{2}$ = 0.50] and was highest immediately after the job interview at time point s_2_ [*t*_(44)_ = 8.37, *p* < 0.001, Cohen's *d* = 1.87; Figure 2C].

### 3.3. Associations with person characteristics

#### 3.3.1. Sex

Furthermore, rmANOVAs for sAA and cortisol with an additional between-subjects factor sex confirmed the above-reported main effect of the factor time [sAA: *F*_(3.56, 146.59)_ = 5.87, *p* < 0.001, $\eta_{\text{p}}^{2}$ = 0.13; cortisol: *F*_(1.57, 65.79)_ = 5.49, *p* = 0.011, $\eta_{\text{p}}^{2}$ = 0.12]. The interaction between the factors time and sex as well as the main effect of sex was not significant for both sAA and cortisol (sAA: *p* > 0.740; cortisol: *p* > 0.106). Therefore, overall, no sex differences were found for sAA and cortisol. For perceived stress, the main effect of time [*F*_(3.55, 152.44)_ = 41.67, *p* < 0.001, $\eta_{\text{p}}^{2}$ = 0.49] and the main effect of the factor sex [*F*_(1, 43)_ = 9.54, *p* = 0.004, $\eta_{\text{p}}^{2}$ = 0.18] were found. The interaction between time and sex was not significant (*p* = 0.028). Perceived stress was higher for female participants than for male participants at the time points s_1_ [*t*_(42.38)_ = 2.68, *p* = 0.005, Cohen's *d* = 1.48], s_3_ (*t*_(42.94)_ = 3.70, *p* < 0.001, Cohen's *d* = 1.74), and s_4_ [*t*_(38.29)_ = 2.44, *p* = 0.010, Cohen's *d* = 1.19; Figure 2D].

#### 3.3.2. Age and BMI

**Figure 3:**
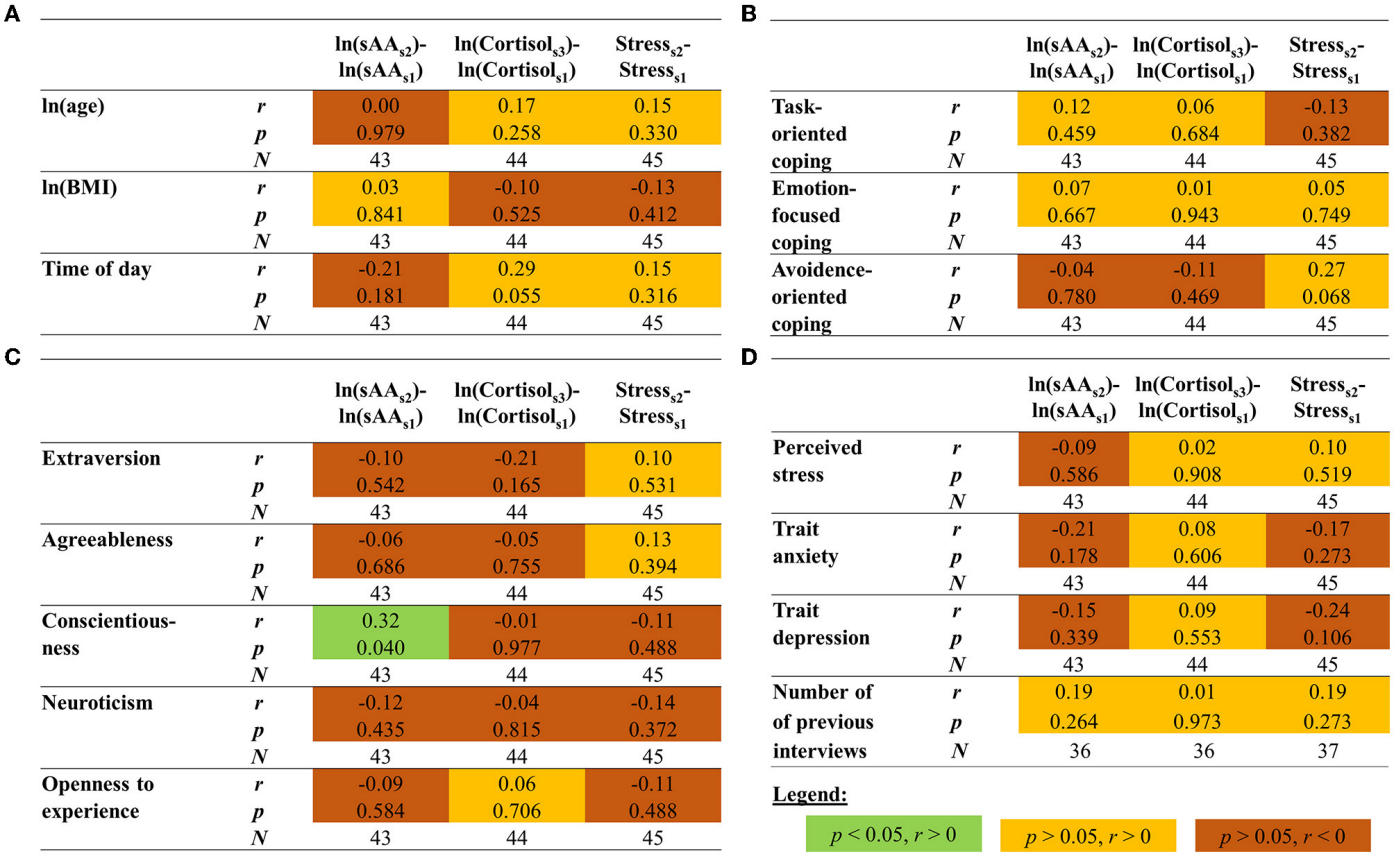
Correlations between the changes in sAA, cortisol, and perceived stress levels and **(A)** age, body mass index (BMI), and time of day, **(B)** coping styles, **(C)** personality traits, and **(D)** perceived stress, trait anxiety, and trait depression, as well as the number of previous job interviews. The correlation with *p* < 0.05 is highlighted in green. Note that this *p*-value was still lower than our adjusted alpha level of.016. Salivary alpha-amylase (sAA) and cortisol levels as well as the variables age and BMI were transformed using the natural logarithm (ln).

Neither age nor BMI was correlated with the change in sAA, cortisol, or perceived stress (all *p* > 0.329; Figure 3A).

#### 3.3.3. Previous job interviews

Most of the participants reported experiences with job interviews (*n* = 37, 82.2%). For those, who had experience, it was investigated whether the stress responses were associated with the number of previous job interviews. No significant correlations were found (all *p* > 0.263; Figure 3D).

### 3.4. Perceiving the situation as a threat or as a challenge

Most of the participants (*n* = 32, 71%) perceived the job interview as a challenge, *n* = 10 (22%) as a threat, and *n* = 3 (7%) neither perceived the situation as a threat nor as a challenge. For sAA, the main effect of the factor time was found again, which reflected the already reported change over time [*F*_(3.50, 133.01)_ = 5.53, *p* < 0.001, $\eta_{\text{p}}^{2}$ = 0.13]. This was not significant for cortisol when using the adjusted alpha level (*p* = 0.048). The interaction between time and threat vs. challenge was not significant for none of the biomarkers (*p* > 0.377). However, the main effect of threat vs. challenge was significant for cortisol [*F*_(1, 39)_ = 7.48, *p* = 0.009, $\eta_{\text{p}}^{2}$ = 0.16], but not for sAA (*p* = 0.205). This reflected higher cortisol levels for the threat group than for the challenge group for the time points s_2_ [*t*_(39)_ = 2.48, *p* = 0.017, Cohen's *d* = 0.66] and s_4_ [*t*_(39)_ = 2.73, *p* = 0.009, Cohen's *d* = 0.66; Figure 2E]. For perceived stress, only the main effect of the factor time was found [*F*_(3.50, 139.95)_ = 30.12, *p* < 0.001, $\eta_{\text{p}}^{2}$ = 0.43], but no significant interaction between the factors time and threat vs. challenge nor the main effect of the factor threat vs. challenge was found (*p* > 0.026).

### 3.5. Associations with psychological variables

None of the psychological variables (i.e., coping styles, personality traits, perceived stress during the last month, state anxiety, and state depression) was significantly related to the increase in sAA, cortisol, or perceived stress (all *p* > 0.019; Figures 3B–D).

### 3.6. Further variables

Another variable, which may be associated with the stress response, is the time of the saliva sampling (i.e., the time of day) because sAA and cortisol levels follow a diurnal rhythm (Nater et al., 2007; Ryan et al., 2016). No significant associations were found between the factor time of day and the increase in sAA, cortisol, or perceived stress (all *p* > 0.054; Figure 3A).

A further factor, which may be associated with the biological variables, is the use of contraceptives (Kirschbaum et al., 1999). Therefore, a sub-analysis was conducted for female participants. Again, only main effects for time were found for sAA and perceived stress [sAA: *F*_(5, 125)_ = 5.96, *p* < 0.001, $\eta_{\text{p}}^{2}$ = 0.19; perceived stress: *F*_(3.20, 86.48)_ = 16.57, *p* < 0.001, $\eta_{\text{p}}^{2}$ = 0.38]. The main effect of time was not significant for cortisol (*p* = 0.223). Neither the interaction time ^*^ use of contraceptives nor the main effect of use of contraceptives were significant for any of the stress markers (all *p* > 0.038).

Finally, it was investigated whether the stress responses differed between participants who had the same sex as the interviewer (*n* = 21, 46.7%) vs. participants with an opponent sex than the interviewer. For sAA and cortisol, only significant effects of the factor time were found [sAA: *F*_(3.58, 146.65)_ = 6.14, *p* < 0.001, $\eta_{\text{p}}^{2}$ = 0.13; cortisol: *F*_(1.64, 68.76)_ = 4.79, *p* = 0.016, $\eta_{\text{p}}^{2}$ = 0.10]. Neither the interaction time ^*^ same vs. not the same sex nor the main effect of same vs. not the same sex was significant (all *p* > 0.048). For perceived stress, the main effect of time [*F*_(3.49, 149.96)_ = 43.96, *p* < 0.001, $\eta_{\text{p}}^{2}$ = 0.51] and the main effect of same vs. not the same sex (*F*_(1, 43)_ = 7.49, *p* = 0.009, $\eta_{\text{p}}^{2}$ = 0.15) were significant, reflecting higher perceived stress for interviewers with the same sex at s_1_ [*t*_(32.48)_ = 3.36, *p* = 0.001, Cohen's *d* = 1.41] and s_4_ [*t*_(43)_ = 2.64, *p* = 0.006, Cohen's *d* = 1.38; Figure 2F]. However, although the effect was significant at s_1_, it should be noted that the interviewer was not known at this time point.

## 4. Discussion

### 4.1. Summary of main findings

The aim of our study was to develop and evaluate a naturalistic-designed stress test, which can be applied in a digital context. We decided to use a digital job interview-scenario. A comprehensive procedure was developed, which lasted about 20–25 min and was individually adopted to the participant, i.e., involving individual questions based on the participant's dream job and his/her strengths and weaknesses. The job interviews triggered typical subjective and biological stress responses, including an increase in perceived stress and sAA levels which peaked immediately after the interviews, as well as an increase in cortisol levels with maximum levels about 5 min later. Sex differences were found for perceived stress, i.e., female participants experienced the scenario as more stressful than male participants. Cortisol levels were higher for participants who experienced the situation as a threat in comparison to participants who experienced it as a challenge.

### 4.2. Discussion of main findings

Overall, our findings support that digital job interviews were associated with biological and subjective stress responses. The time point of the maximum levels for sAA and perceived stress (i.e., immediately after the job interview) was expected regarding the fast time course of SNS responses. However, peak cortisol levels already occurred 5 min later, which was earlier than expected based on our experience with further stressors and which are usually reported (e.g., Becker and Rohleder, 2019). One reason may be that the participants were already stressed in the anticipation of the stressor. The relatively high baseline levels are in favor of this explanation. This is also one important difference between our stress test and typical laboratory stressors that can be found in the literature. Our participants knew in advance that they will take part in a selection interview and had the chance (although this was not introduced) to prepare themselves for the appointment. Furthermore, the interviews which were conducted after the job interviews may have altered the HPA axis response and may be another reason for the untypical cortisol time course.

Sex differences in stress responses have also been reported previously. Usually, stronger HPA axis responses are found for male participants than for female participants (Kudielka et al., 2009). This effect was not significant in our study, but on a descriptive level, cortisol levels pointed in the same direction (i.e., they were higher for male than for female participants). Our finding of higher perceived stress during the interviews for female participants may be affected by social desirability. Social-desirability bias is gender-specific and has been found to be stronger in female participants than in male participants (Chung and Monroe, 2003; Dalton and Ortegren, 2011).

In TSST studies (Kirschbaum et al., 1993), it has been found that stronger responses occur in the presence of an opponent sex experimenter (Goodman et al., 2017). In our study, we found an association in the opposite direction with higher perceived stress levels when the experimenter (i.e., the interviewer) had the same sex as the participant. Again, this finding may be biased by social desirability. Moreover, although our scenario has some similarities with the TSST (which also includes a presentation of the participant in front of a jury, which is a scenario similar to job interviews), there are some differences. The job interview-scenario in our study was more naturalistic and more specifically adjusted to the individual participant than in typical TSST studies or other standardized stress tests.

Our study also supports the well-known finding that especially threatening rather than challenging stressors lead to strong biological stress responses (Kemeny, 2003). Appraisal processes are associated with coping styles (Folkman, 2020). Associations between stress responses and coping have been reported previously (Bohnen et al., 1991; Janson and Rohleder, 2017), which could not be supported by our data. We conclude that for our scenario, the threat vs. challenge distinction was the more appropriate measure than the distinction between task-oriented, emotion-focused, and avoidance-oriented coping styles.

Moreover, we tested whether the strength of the stress response is associated with personality traits, but we did not find any association. Recent research has highlighted the importance of the personality traits extraversion and neuroticism in the context of the stress response (Vollrath, 2001). One reason that we could not support this assumption may be that the association has been masked by a third factor such as self-efficacy (Ebstrup et al., 2011), which we did not assess in our study. Further research is needed to fully understand these important relations, and data from several studies should be aggregated (Pruessner et al., 1997).

### 4.3. Limitations and future research

One limitation of our study is the mean age of our participants who were young adults in most cases. Data from older participants had to be excluded from analysis based on statistical criteria although we did not make any restrictions on participants' age during the selection process. However, in future research, our scenario should be evaluated in an older sample. Furthermore, the level of education was overall high, and future research should focus on a broader range of educational levels and occupations. Moreover, all participants were either employees or students and, therefore, the procedure must be evaluated in an unemployed sample. Additionally, further factors, which may be associated with the stress response such as the participants' cognitive functioning (Grimm et al., 2021) or self-efficacy (Ebstrup et al., 2011), should be assessed in future studies. Despite these limitations, the characteristics of participants who took part in our study is comparable to populations that have been evaluated in typical laboratory studies on acute stress responses, in which typical stress tests have been used (Supplementary material 2).

In this article, we reported analyses with respect to the most important outcome variables which are usually reported in research on acute stress responses (i.e., sAA as a SNS measure, cortisol as a marker for HPA axis activity, and perceived stress levels as a marker for subjective stress perception). However, as described in the Introduction (1), the acute stress response is complex, and a variety of stress measures is conceivable (see Becker et al., 2022a for an overview). Besides the outcomes, that we reported here, we collected and analyzed further stress measures such as heart rate, movements, and pupil size (Heimerl et al., 2022a), and used standardized interviews to assess participants' emotions during the job interviews (Heimerl et al., 2023). Therefore, we have already covert a wide range of measures. Merely, it was not possible to collect measures of immune system activity, which reflects a further, much slower reactive, biological stress system. However, especially in the working context, this may be important for employees' long-term health (Kaltenegger et al., 2021, 2023).

Another direction for future research is to investigate whether the same stress response patterns can be found in a similar, but non-digital scenario. Besides the further evaluation of the here proposed method as a digital stress test, the application could be used for further purposes such as being the basis of job interview trainings, which can also be designed as digital applications (Gebhard et al., 2014). Overall, job interview trainings are important because stress during the selection process can negatively impact the outcome (Campion et al., 1997). Our scenario may be well-suited for practicing this important situation. Virtual job interview-trainings can increase performance during the interview, and may therefore increase hiring chances (Smith et al., 2021). A first prototype which is based on our job interview-scenario has already been developed in our group (Heimerl et al., 2022b). In future studies, this system will be further developed and evaluated (e.g., with respect to perceived stress and biological stress responses).

## 5. Conclusion

Overall, our digital job interview-scenario is very well-suited to induce biological and perceived stress responses, mostly independent of person characteristics and psychological variables such as personality traits and coping styles. Therefore, it can be applied to a variety of different people. All materials, including the specific questions that were used during the job interviews as well as the rating scales, are freely available (https://osf.io/5bdyf/ and S1), and future research can build on it.

## Data availability statement

The datasets presented in this study can be found in online repositories. The names of the repository/repositories and accession number(s) can be found below: Open Science Framework: https://osf.io/5bdyf/.

## Ethics statement

The studies involving human participants were reviewed and approved by Ethics Committee of the Medical Faculty of the Friedrich-Alexander-Universität Erlangen-Nürnberg (Krankenhausstraße 12, 91054 Erlangen, Germany). The participants provided their written informed consent to participate in this study.

## Author contributions

LB and AH designed and conducted the study and supervised data collection. LB analyzed the data and wrote the manuscript. AH and EA provided critical feedback on the manuscript. LB and EA received funding for this manuscript. All authors read and approved the final version of the manuscript.

## Supplementary material

Supplementary material 1 Questions during the different parts of the job interviews.

Supplementary material 2 Comparison of sample characteristics with those of previous studies.

## References

[B1] Becker L. Kaltenegger H. C. Nowak D. Rohleder N. Weigl M. (2022a). Differences in stress system (re-)activity between single and dual- or multitasking in healthy adults: a systematic review and meta-analysis. Health Psychol. Rev. 17, 78–103. 10.1080/17437199.2022.2071323 35477383

[B2] Becker L. Kaltenegger H. C. Nowak D. Weigl M. Rohleder N. (2022b). Physiological stress in response to multitasking and work interruptions: Study protocol. PLoS ONE 17:e0263785. 10.1371/journal.pone.0263785 35134093 PMC8824354

[B3] Becker L. Rohleder N. (2019). Time course of the physiological stress response to an acute stressor and its associations with the primacy and recency effect of the serial position curve. PLoS ONE 14:e0213883. 10.1371/journal.pone.0213883 31100063 PMC6524805

[B4] Becker L. Rohleder N. (2020). Associations between attention and implicit associative learning in healthy adults: the role of cortisol and salivary alpha-amylase responses to an acute stressor. Brain Sci. 10:544. 10.3390/brainsci10080544 32806521 PMC7463622

[B5] Becker L. Rohleder N. Rohleder O. (In press). “Salivary hormone assays,” in APA Handbook Of Research Methods, Second Edition, eds H. Cooper.

[B6] Becker L. Schade U. Rohleder N. (2019). Evaluation of the socially evaluated cold-pressor group test (SECPT-G) in the general population. PeerJ 7:e7521. 10.7717/peerj.7521 31423367 PMC6697040

[B7] Bohnen N. Nicolson N. Sulon J. Jolles J. (1991). Coping style, trait anxiety and cortisol reactivity during mental stress. J. Psychosom. Res. 35, 141–147. 10.1016/0022-3999(91)90068-Y 2046048

[B8] Bosch J. A. Geus E. J. C. de Veerman, E. C. I. Hoogstraten J. Amerongen A. V. N. (2003). Innate secretory immunity in response to laboratory stressors that evoke distinct patterns of cardiac autonomic activity. Psychosom. Med. 65, 245–258. 10.1097/01.PSY.0000058376.50240.2D 12651992

[B9] Budnick C. J. Anderson E. M. Santuzzi A. M. Grippo A. J. Matuszewich L. (2019). Social anxiety and employment interviews: does nonverbal feedback differentially predict cortisol and performance? Anxiety Stress Coping 32, 67–81. 10.1080/10615806.2018.1530349 30298757

[B10] Campion M. A. Palmer D. K. Campion J. E. (1997). A review of structure in the selection interview. Pers. Psychol. 50, 655–702. 10.1111/j.1744-6570.1997.tb00709.x

[B11] Chung J. Monroe G. S. (2003). Exploring social desirability bias. J. Bus. Ethics 44, 291–302. 10.1023/A:1023648703356

[B12] Cohen S. Kamarck T. Mermelstein R. (1994). Perceived stress scale. Measuring Stress: A Guide for Health and Social Scientists, 235–283.

[B13] Dalton D. Ortegren M. (2011). Gender differences in ethics research: the importance of controlling for the social desirability response bias. J. Bus. Ethics 103, 73–93. 10.1007/s10551-011-0843-8

[B14] Dedovic K. Renwick R. Mahani N. K. Engert V. Lupien S. J. Pruessner J. C. (2005). The montreal imaging stress task: using functional imaging to investigate the effects of perceiving and processing psychosocial stress in the human brain. J. Psychiatry Neurosci. 30, 319–325. 16151536 PMC1197276

[B15] Ebstrup J. F. Eplov L. F. Pisinger C. Jørgensen T. (2011). Association between the five factor personality traits and perceived stress: is the effect mediated by general self-efficacy? Anxiety Stress Coping 24, 407–419. 10.1080/10615806.2010.540012 21213153

[B16] Endler N. Parker J. D. A. (2008). Coping Inventory for Stressful Situations.

[B17] Folkman S. (2020). Stress: Appraisal and Coping. In Encyclopedia of Behavioral Medicine. ed M. D. Gellman (Cham: Springer). 10.1007/978-3-030-39903-0_215

[B18] Fulford A. J. Harbuz M. S. (2005). An introduction to the HPA axis. In Techniques in the Behavioral and Neural Sciences. Elsevier, 43–65. 10.1016/S0921-0709(05)80006-9

[B19] Gebhard P. Baur T. Damian I. Mehlmann G. Wagner J. André E. (2014). Exploring Interaction Strategies for Virtual Characters to Induce Stress in Simulated Job Interviews.

[B20] Goodman W. K. Janson J. Wolf J. M. (2017). Meta-analytical assessment of the effects of protocol variations on cortisol responses to the Trier Social Stress Test. Psychoneuroendocrinology 80, 26–35. 10.1016/j.psyneuen.2017.02.030 28292684

[B21] Greenhouse S. W. Geisser S. (1959). On methods in the analysis of profile data. Psychometrika 24, 95–112. 10.1007/BF02289823

[B22] Grimm E. Agrigoroaei S. Rohleder N. Becker L. (2021). Executive Functioning as a predictor of physiological and subjective acute stress responses in non-clinical adult populations: a systematic literature review and meta-analysis. Neurosci. Biobehav. Rev. 131:1096–1115. 10.1016/j.neubiorev.2021.09.037 34562543

[B23] Heimerl A. Becker L. Schiller D. Baur T. Wildgrube F. Rohleder N.. (2022a). “We've never been eye to eye: a pupillometry pipeline for the detection of stress and negative affect in remote working scenarios,” in Proceedings of the 15^*th*^ International Conference on PErvasive Technologies Related to Assistive Environments, 486–493. 10.1145/3529190.3534729

[B24] Heimerl A. Mertes S. Schneeberger T. Baur T. Liu A. Becker L.. (2022b). “Generating personalized behavioral feedback for a virtual job interview training system through adversarial learning,” in Lecture Notes in Computer Science: Vol. 13355. Artificial intelligence in education: 23^*rd*^ *International Conference, AIED 2022*, eds M. M. Rodrigo, N. Matsuda, A. I. Cristea, and V. Dimitrova, (Durham: Springer), 679–684. 10.1007/978-3-031-11644-5_67

[B25] Heimerl A. Prajod P. Mertes S. Baur T. Kraus M. Liu A.. (2023). ForDigitStress: A multi-modal stress dataset employing a digital job interview scenario. *arXiv [Preprint]. arXiv: 2303.07742*. Available online at: https://arxiv.org/pdf/2303.07742.pdf

[B26] Janson J. Rohleder N. (2017). Distraction coping predicts better cortisol recovery after acute psychosocial stress. Biol. Psychol. 128, 117–124. 10.1016/j.biopsycho.2017.07.014 28743456

[B27] Kälin W. Semmer N. (2020). Coping-Inventar zum Umgang mit Stress-Situationen: Deutschsprachige Adaptation des Coping Inventory for Stressful Situations (CISS) von Norman S. Endler und James D. A. Parker. Hogrefe.

[B28] Kaltenegger H. C. Becker L. Rohleder N. Nowak D. Quartucci C. Weigl M. (2023). Associations of techno-stressors at work with burnout symptoms and chronic low-grade inflammation: a cross-sectional analysis in hospital employees. Int. Arch. Occup. Environ. Health, 1–18. 10.1007/s00420-023-01967-8 37148328 PMC10163295

[B29] Kaltenegger H. C. Becker L. Rohleder N. Nowak D. Weigl M. (2021). Associations of working conditions and chronic low-grade inflammation among employees: a systematic review and meta-analysis. Scan. J. Work Environ. Health 47, 565–581. 10.5271/sjweh.3982 34523689 PMC9058622

[B30] Kemeny M. E. (2003). The psychobiology of stress. Curr. Dir. Psychol. Sci. 12, 124–129. 10.1111/1467-8721.01246

[B31] Kirschbaum C. Kudielka B. M. Gaab J. Schommer N. C. Hellhammer D. H. (1999). Impact of gender, menstrual cycle phase, and oral contraceptives on the activity of the hypothalamus-pituitary-adrenal axis. Psychosom. Med. 61, 154–162. 10.1097/00006842-199903000-00006 10204967

[B32] Kirschbaum C. Pirke K. M. Hellhammer D. H. (1993). The ‘trier social stress test'–a tool for investigating psychobiological stress responses in a laboratory setting. Neuropsychobiology 28, 76–81. 10.1159/000119004 8255414

[B33] Klein E. M. Brähler E. Dreier M. Reinecke L. Müller K. W. Schmutzer G.. (2016). The German version of the Perceived Stress Scale–psychometric characteristics in a representative German community sample. BMC Psychiatry 16, 159. 10.1186/s12888-016-0875-9 27216151 PMC4877813

[B34] Kudielka B. M. Hellhammer D. H. Wüst S. (2009). Why do we respond so differently? reviewing determinants of human salivary cortisol responses to challenge. Psychoneuroendocrinology 34, 2–18. 10.1016/j.psyneuen.2008.10.004 19041187

[B35] Laux L. Hock M. Bergner-Köther R. Hodapp V. Renner K. -H. (2013). Das State-Trait-Angst-Depressions-Inventar: STADI, Manual.

[B36] Lazarus R. S. Folkman S. (1984). Stress, Appraisal, and Coping. Springer Publishing Company.

[B37] Lazarus R. S. Folkman S. (1987). Transactional theory and research on emotions and coping. Eur. J. Pers. 1, 141–169. 10.1002/per.2410010304

[B38] Mauchly J. W. (1940). Significance test for sphericity of a normal n-variate distribution. Ann. Math. Stat. 11, 204–209. 10.1214/aoms/1177731915

[B39] Nater U. M. Rohleder N. (2009). Salivary alpha-amylase as a non-invasive biomarker for the sympathetic nervous system: current state of research. Psychoneuroendocrinology 34, 486–496. 10.1016/j.psyneuen.2009.01.014 19249160

[B40] Nater U. M. Rohleder N. Schlotz W. Ehlert U. Kirschbaum C. (2007). Determinants of the diurnal course of salivary alpha-amylase. Psychoneuroendocrinology 32, 392–401. 10.1016/j.psyneuen.2007.02.007 17418498

[B41] Pruessner J. C. Gaab J. Hellhammer D. H. Lintz D. Schommer N. Kirschbaum C. (1997). Increasing correlations between personality traits and cortisol stress responses obtained by data aggregation. Psychoneuroendocrinology 22, 615–625. 10.1016/S0306-4530(97)00072-3 9483706

[B42] Putignano P. Dubini A. Toja P. Invitti C. Bonfanti S. Redaelli G.. (2001). Salivary cortisol measurement in normal-weight, obese and anorexic women: comparison with plasma cortisol. Eur. J. Endocrinol. 145, 165–171. 10.1530/eje.0.1450165 11454512

[B43] Rammstedt B. John O. P. (2005). Kurzversion des big five inventory (BFI-K). Diagnostica 51, 195–206. 10.1026/0012-1924.51.4.195

[B44] Ryan R. Booth S. Spathis A. Mollart S. Clow A. (2016). Use of salivary diurnal cortisol as an outcome measure in randomised controlled trials: a systematic review. Ann. Behav. Med. 50, 210–236. 10.1007/s12160-015-9753-9 27007274 PMC4823366

[B45] Schwabe L. Haddad L. Schachinger H. (2008). HPA axis activation by a socially evaluated cold-pressor test. Psychoneuroendocrinology 33, 890–895. 10.1016/j.psyneuen.2008.03.001 18403130

[B46] Selye H. (1950). Stress and the general adaptation syndrome. Br. Med. J. 1:1383. 10.1136/bmj.1.4667.1383 15426759 PMC2038162

[B47] Smith M. J. Smith J. D. Jordan N. Sherwood K. McRobert E. Ross B.. (2021). Virtual reality job interview training in transition services: results of a single-arm, noncontrolled effectiveness-implementation hybrid trial. J. Spec. Educ. Technol. 36, 3–17. 10.1177/0162643420960093 PMC11192452 38911489

[B48] Vining R. F. McGinley R. A. Maksvytis J. J. Ho K. Y. (1983). Salivary cortisol: a better measure of adrenal cortical function than serum cortisol. Ann. Clin. Biochem. 20, 329–335. 10.1177/000456328302000601 6316831

[B49] Vollrath M. (2001). Personality and stress. Scan. J. Psychol. 42, 335–347. 10.1111/1467-9450.00245 11547909

