# Supplementary material for: ForDigitStress: presentation and evaluation of a new laboratory stressor using a digital job interview-scenario
Source: Front Psychol. 2023 Jun 19;14:1182959. doi: 10.3389/fpsyg.2023.1182959 (PMC10315916; doi:10.3389/fpsyg.2023.1182959)
Supplement: Supplementary material 1 — Questions during the different parts of the job interviews. [file Data_Sheet_1.pdf]

Supplementary Material 1

Becker, L., Heimerl, A., & André, E. (2023)

ForDigitStress: Questions during the job interviews

The job-interviews were semi-standardized, i.e., a typical protocol was followed, which was adopted to the individual participant. Not every part and not every question was used for every participant. The interviews lasted about 20 – 25 minutes. The language of the original interviews was German.

1) Welcome and introduction

- Welcome to the selection interview at company … for the position as …
- …

2) Introduction of the applicant

- Please introduce yourself.
- How would you describe yourself?
- Who are you?
- …

3) Interruption of the introduction

- Don't tell me anything that I could read on your CV. Go on with describing/introducing yourself.

4) Questions regarding the employer/company

- How did you find out about the position?
- Why did you apply for the position?
- Why do you find the job offer interesting?
- Why do you want to join this company?
- What do you know about our company?
- …

5) Job change-explanation

- Why are you looking for a new job?
- Were there any conflicts during your last job.
- What would you do differently than your last boss?

6) Expectations of the applicant

- What do you expect from the job?
- What are your hopes regarding this job?
- …

7) Advertising of the applicant

- What qualifies you for this job?
- What are your greatest strengths and weaknesses?
- Why should we choose you?
- What qualifies you?
- …

8) Hypothetical situation

- How would you react if...?
  - … your colleague embarrasses you in a meeting?
  - …your manager tells you that your work is not satisfactory?
  - … a patient collapses next to you and is no longer responsive?
- Your application states that you own a driver's license. Then this is what you need to know… patient refuses to talk to you? …
- …

9) Applicant's future vision

- Where do you see yourself in 5 years?
- …

10) Practical task

- Do you have a pen? [a pen lies in front of the participant]
  - Sell me this pen.
- …

11) Basic knowledge questions

- Which state do the Phoenix Islands belong to? [Kiribati]
- Where are the Cayman Islands located? [South of Cuba]
- Who is the chemist behind the current periodic table? [Dmitri Mendeleev]
- How many stars does the European flag have? [12]
- What is the name of a work by the German writer Günter Grass? [The flounder]
- Who elects the Federal President? [Federal Assembly]
- On what date did the Berlin Wall fall? [October 8, 1989]
- …

12) Subject-specific questions

Basic knowledge psychology:

- Who wrote the book "Critique of Pure Reason"? [Immanuel Kant]
- What is psychotherapy?
- What are the ICD-10 criteria for unipolar depression? How do they differ from the DSM-V criteria?
- After September 11, 2001, many people said that "American intelligence agencies should have foreseen the likelihood of this form of terrorism". What does this perception clearly demonstrate? [Hindsight bias]
- …

Basic-knowledge language:

- Please translate the following sentence into…: "Ein guter Koch könnte so viele Kekse kochen, wie ein guter Koch, der Kekse kochen könnte".
- …

Basic-knowledge mathematics and puzzles:

- How often does a stopped clock show the correct time during the day?
- A jeweler has a serious luxury problem. He has 27 completely identical looking beads that he wants to use to make a necklace. However, he knows that exactly one of these 27 pearls is

wrong. - He also knows from experience that a fake pearl is always a bit heavier than a real one. Unfortunately, for reasons of tradition, the jeweler has one pan scale. He doesn't own the weights. What is the minimum number of times the jeweler has to weigh to find out which of the 27 pearls is the wrong one?

- Please do the math:
  - 112:6 = …
  - 13:5 = …
  - Calculate the checksum of…
- …

13) Questions regarding the applicant's outfit

- Why are you wearing this outfit today?
- …

14) Ideas regarding salary and working hours

- How much do you want to earn?
- How many hours a week would you like to work?
- Are you flexible?
- Do you sometimes jump in spontaneously?
- Are you willing to work overtime?
- …

15) End

- Do you have any more questions?
- We'll get in touch with you, but we have further applicants.
