# Supplementary material for: ForDigitStress: presentation and evaluation of a new laboratory stressor using a digital job interview-scenario
Source: Front Psychol. 2023 Jun 19;14:1182959. doi: 10.3389/fpsyg.2023.1182959 (PMC10315916; doi:10.3389/fpsyg.2023.1182959)
Supplement: Supplementary material 2 — Comparison of sample characteristics with those of previous studies. [file Data_Sheet_2.pdf]

Supplementary Material 2

Becker, L., Heimerl, A., & André, E. (2023)

ForDigitStress:
Comparison of sample characteristics with those of previous studies

Study	Stress task	*N*	%female	Mean age (years)	Standard deviation age (years)	Range age (years)
**Present study: Becker et al. (2023)**	**ForDigitStress (digital job interviews)**	**45**	**64.4**	**23.2**	**3.6**	**18 – 33**
Becker and Rohleder (2019)	SECPT	33	75.8	24.0	5.7	NR
Becker and Rohleder (2020)	SECPT	81	69.1	21.8	3.9	NR
Dedovic et al. (2005), study 1	MIST	10	10	NR	NR	21 – 30
Dedovic et al. (2005), study 2	MIST	10	0	NR	NR	20 – 25
Janson and Rohleder (2017)	TSST	61	60.7	22.9	4.3	NR
Kirschbaum et al. (1993), study 1	TSST	20	0	24.7	3.3	NR
Kirschbaum et al. (1993), further studies	TSST	155	Both sexes, not further specified	NR	NR	15 – 33
Kirschbaum et al. (1999)	TSST	81	75.3	22.8 – 23.7 in dependence on sex and menstrual cycle	0.4 – 0.8 in dependence on sex and menstrual cycle	18 – 32
Schwabe et al. (2008)	SECPT	70	0	23.7	2.9	19 – 35
Wiemers et al. (2013)	TSST and friendly version of the TSST	48	50	23.9	2.4	19 – 30

Note. SECPT = socially evaluative cold-pressor test; MIST = Montreal Imaging Stress Task; TSST = Trier Social Stress Test; NR = not reported.

ForDigitStress (Becker et al., 2023)

References

Becker, L., & Rohleder, N. (2019). Time course of the physiological stress response to an acute stressor and its associations with the primacy and recency effect of the serial position curve. *PloS One*, *14*(5), e0213883.

Becker, L., & Rohleder, N. (2020). Associations between Attention and Implicit Associative Learning in Healthy Adults: The Role of Cortisol and Salivary Alpha-Amylase Responses to an Acute Stressor. *Brain Sciences*, *10*(8), 544.

Becker, L., Schade, U., & Rohleder, N. (2019). Evaluation of the socially evaluated cold-pressor group test (SECPT-G) in the general population. *PeerJ*, *7*, e7521.

Dedovic, K., Renwick, R., Mahani, N. K., Engert, V., Lupien, S. J., & Pruessner, J. C. (2005). The Montreal Imaging Stress Task: using functional imaging to investigate the effects of perceiving and processing psychosocial stress in the human brain. *Journal of Psychiatry and Neuroscience*, *30*(5), 319.

Janson, J., & Rohleder, N. (2017). Distraction coping predicts better cortisol recovery after acute psychosocial stress. *Biological Psychology*, *128*, 117–124.

Kirschbaum, C., Kudielka, B. M., Gaab, J., Schommer, N. C., & Hellhammer, D. H. (1999). Impact of gender, menstrual cycle phase, and oral contraceptives on the activity of the hypothalamus-pituitary-adrenal axis. *Psychosomatic Medicine*, *61*(2), 154–162.

Kirschbaum, Pirke, & Hellhammer (1993). The 'Trier Social Stress Test'–a tool for investigating psychobiological stress responses in a laboratory setting. *Neuropsychobiology*, *28*(1-2), 76–81.

Schwabe, L., Haddad, L., & Schachinger, H. (2008). HPA axis activation by a socially evaluated cold-pressor test. *Psychoneuroendocrinology*, *33*(6), 890–895.

Wiemers, U. S., Schoofs, D., & Wolf, O. T. (2013). A friendly version of the Trier Social Stress Test does not activate the HPA axis in healthy men and women. *Stress*, *16*(2), 254-260.
